# PD‐L1 regulates tumor proliferation and T‐cell function in NF2‐associated meningiomas

**DOI:** 10.1111/cns.14784

**Published:** 2024-06-03

**Authors:** Ying Wang, Chao Zhang, Minjun Yan, Xin Ma, Lairong Song, Bo Wang, Peng Li, Pinan Liu

**Affiliations:** ^1^ Beijing Neurosurgical Institute Capital Medical University Beijing China; ^2^ Department of Neurosurgery, Beijing Tiantan Hospital Capital Medical University Beijing China

**Keywords:** immunosuppression, meningioma, NF2, PDL1, PI3K/AKT/mTOR

## Abstract

**Introduction:**

Programmed death‐ligand 1 (PD‐L1) expression is an immune evasion mechanism that has been demonstrated in many tumors and is commonly associated with a poor prognosis. Over the years, anti‐PD‐L1 agents have gained attention as novel anticancer therapeutics that induce durable tumor regression in numerous malignancies. They may be a new treatment choice for neurofibromatosis type 2 (NF2) patients.

**Aims:**

The aims of this study were to detect the expression of PD‐L1 in NF2‐associated meningiomas, explore the effect of PD‐L1 downregulation on tumor cell characteristics and T‐cell functions, and investigate the possible pathways that regulate PD‐L1 expression to further dissect the possible mechanism of immune suppression in NF2 tumors and to provide new treatment options for NF2 patients.

**Results:**

PD‐L1 is heterogeneously expressed in NF2‐associated meningiomas. After PD‐L1 knockdown in NF2‐associated meningioma cells, tumor cell proliferation was significantly inhibited, and the apoptosis rate was elevated. When T cells were cocultured with siPD‐L1‐transfected NF2‐associated meningioma cells, the expression of CD69 on both CD4^+^ and CD8^+^ T cells was partly reversed, and the capacity of CD8^+^ T cells to kill siPD‐L1‐transfected tumor cells was partly restored. Results also showed that the PI3K–AKT–mTOR pathway regulates PD‐L1 expression, and the mTOR inhibitor rapamycin rapidly and persistently suppresses PD‐L1 expression. In vivo experimental results suggested that anti‐PD‐L1 antibody may have a synergetic effect with the mTOR inhibitor in reducing tumor cell proliferation and that reduced PD‐L1 expression could contribute to antitumor efficacy.

**Conclusions:**

Targeting PD‐L1 could be helpful for restoring the function of tumor‐infiltrating lymphocytes and inducing apoptosis to inhibit tumor proliferation in NF2‐associated meningiomas. Dissecting the mechanisms of the PD‐L1‐driven tumorigenesis of NF2‐associated meningioma will help to improve our understanding of the mechanisms underlying tumor progression and could facilitate further refinement of current therapies to improve the treatment of NF2 patients.

## Graphical abstract

Positive PDL1 expression was observed in NF2‐meningiomas, downregulation of PD‐L1 could inhibit tumor cell proliferation, promote apoptosis, and suppress the inhibitory effect of tumor cells on T‐cell activation and cytotoxicity. NF2 may regulate PD‐L1 expression through the PI3K–AKT–mTOR pathway, anti‐PD‐L1 antibody may have a synergetic effect with the mTOR inhibitor in reducing tumor cell proliferation.

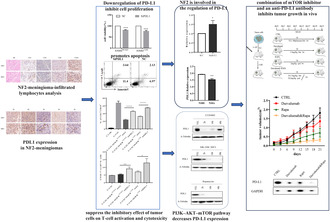

## 1 INTRODUCTION

Neurofibromatosis type 2 (NF2) is an autosomal dominant disorder with characteristic bilateral vestibular schwannomas,^1^ and up to 80% of NF2 patients eventually develop meningiomas.^2^ Meningiomas, as the second most common tumor type of NF2, often include multiple lesions and develop in young patients.^3^ NF2‐associated meningiomas frequently show aggressive clinical features and can cause serious neurological deficits. NF2 patients can develop new meningiomas throughout their lives and often need multiple surgeries to resect these tumors.^4^ Some patients have too many meningiomas and lose the chance to receive surgical treatment. NF2‐associated meningiomas are often associated with a poor prognosis, with a 2.5‐fold increase in the relative mortality rate compared to that for the condition as a whole.^5^ Thus, to improve patient prognosis, there is an urgent need to study the biological features of NF2‐associated meningiomas and find a new effective treatment method.

In recent years, immunotherapy has become a major tumor treatment focus, as it holds the potential to induce durable responses, provides insights for improving patients' quality of life and prolongs the overall survival (OS) of patients.^6^, ^7^ We have been curious about the function of immunotherapy in NF2 patients for a long time. In previous research,^8^ we found that NF2 patients present an immunosuppressive state and that myeloid‐derived suppressor cells (MDSCs), characterized by the HLA‐DR^−^CD33^+^CD11b^+^ phenotype, mediate immunosuppressive function. We also demonstrated that the application of specific neutralizing antibodies to block the function of TGF‐β could partly reduce MDSC‐mediated inhibition of T cells, indicating that elucidation of such NF2‐associated tumor‐initiated suppressive mechanisms could be helpful for designing effective immunotherapeutic protocols and likely bring new possible therapeutic interventions for NF2 patients.

More recently, immune response stimulation with monoclonal antibodies targeting immune checkpoints (known as immune checkpoint inhibitors, ICIs) has revolutionized cancer treatment, which has yielded an increase in overall survival in many tumor settings.^9^, ^10^, ^11^ PD‐L1 is mainly expressed on the surface of tumor cells and antigen‐presenting cells and binds to PD‐1 to perform its immunosuppressive function.^12^ At present, it is believed that PD‐L1 may serve as a biomarker of potential clinical benefits, but not all tumors with high expression of PD‐L1 have a significant response to a PD‐L1/PD‐1 inhibitor, and some tumor patients with PD‐L1‐negative tumors can still have a significant response to a PD‐L1/PD‐1 inhibitor.^13^, ^14^ We are curious about how NF2‐associated meningiomas respond to PD‐L1‐targeting therapy.

In this study, we detected the expression of PD‐L1 in NF2‐associated meningiomas, explored the effect of PD‐L1 downregulation on tumor cell characteristics and T‐cell functions, and investigated the possible pathways that regulate PD‐L1 expression to further dissect the possible mechanism of immune suppression in NF2 tumors and to provide new treatment options for NF2 patients.

## 2 MATERIALS AND METHODS

### 2.1 Patients

For all procedures relating to the human body, the ethical guidelines of the organization and/or the National Scientific Research Council were followed, and these procedures comply with the 1964 Helsinki Declaration and subsequent revisions or similar guidelines. Our study was approved by the medical management department of Beijing Tiantan Hospital, and consent was obtained from all patients before participating. All the specimens of the patients were associated with NF2 and were diagnosed by neuropathologists at Beijing Tiantan Hospital. A total of 13 NF2 patients were enrolled for this project. Patients fulfilled the Manchester diagnostic criteria for NF2, had bilateral acoustic neuroma and meningiomas, and were not treated with drugs or radiation therapy.

Details of NF2 patients are described in Table 1.

**TABLE 1:** Details of NF2 patients.

Details of NF2 patients	Numbers (%)
All patients (*n* = 13)	
Median age	36 ± 16.1
Gender	
Male	7 (53.85%)
Female	6 (46.15%)

### 2.2 Immunohistochemistry (IHC)

Fresh NF2‐associated meningioma tissues were fixed in 4% paraformaldehyde, dehydrated in ethanol, embedded in paraffin, sectioned at 5 μm thickness, and then subjected to deparaffinization, hydration, blocking and peroxide treatment. Following this, primary antibodies were incubated overnight, washed with PBS, incubated with secondary antibody for 1 h, and treated with DAB solution. Finally, tissues were counterstained with hematoxylin and dehydrated. A light microscope was used (Olympus Corporation, Tokyo, Japan) at 200×/400× magnification.

### 2.3 Establishment of NF2‐associated meningioma cell lines

IOMM‐Lee cells were infected with NF2 shRNA/NF2‐518A/NF2‐518D lenti‐GFP virus. These lentiviruses were applied to cells at a multiplicity of infection (MOI) of 50, and then 4 weeks of drug selection with puromycin (5 μg/mL) was performed to obtain stable NF2‐associated meningioma cell lines (IOMM^NF2KD^, IOMM^518A^, IOMM^518D^). NF2 knockdown was confirmed using RT–PCR and Western blot analysis.

### 2.4 Transfection

NF2‐associated meningioma cells (IOMM^NF2KD^, IOMM^518D^) were transfected with a pool of 3 PD‐L1 siRNAs or scrambled siRNAs (RIBOBio Co., Ltd., Guangzhou, China). PD‐L1 knockdown was confirmed using RT–PCR and Western blot analysis.

### 2.5 Cell viability assay

After 72 h of transfection with PD‐L1 siRNA, NF2‐associated meningioma cells (IOMM^NF2KD^, IOMM^518D^) were seeded in 96‐well plates (*n* = 3). Twenty‐four hours later, CCK‐8 was added to each well and cultured for 2 h at 37°C. Then, using SpectraMax M5 (Molecular Devices, Sunnyvale, CA, USA), the intensity of the color developed was measured at 450 nm. The cell viability was expressed as the percentage of control cells cultured in transfected with scrambled siRNA, which was set to 100%.

### 2.6 Apoptosis assay

Apoptotic cells were determined by the Annexin V‐FITC/7‐AAD Kit (BD, CA, USA). After 72 h of transfection with PD‐L1 siRNA or scrambled siRNA at a density of 3 × 10^5^ cells/well in six‐well plates, the fluorescence intensities were measured using flow cytometry (BD, CA, USA).

### 2.7 T‐cell activation and cytotoxicity assay

CD3^+^ T lymphocytes were acquired from the peripheral blood of healthy donors using a CD3 T Kit with MACS (Miltenyi Biotech, Bergisch Gladbach, Germany). Experiments were carried out according to the manufacturer's instructions. CD3^+^ T cells were suspended and incubated with (1) PBS, (2) Cell Stimulation Cocktail (500×, eBioscience™, CSC), (3) CSC + IOMM^NF2KD^‐SiNC, (4) CSC + IOMM^NF2KD^‐SiPD‐L1, (5) CSC + IOMM^518D^‐SiNC, and (6) CSC + IOMM^518D^‐SiPD‐L1. After 24 h, the CD3^+^ T cells were harvested, stained with CD4‐PE‐CY7, CD8‐PE, and CD69‐FITC, and then fixed and permeabilized. T‐cell activation rates were determined by the percentage of CD69^+^ cells in gated CD4^+^ or CD8^+^ cells.

For the cytotoxicity assay, NF2‐associated meningioma cells (IOMM^NF2KD^, IOMM^518D^) were stained with CFSE (1.5 μM, Molecular Probe/Invitrogen), and responder CD8^+^ T cells were isolated from healthy donors using a CD8 T Kit with MACS (Miltenyi Biotech, Bergisch Gladbach, Germany), stimulated with a T‐cell activation and expansion kit (Miltenyi Biotech) and then cocultured with NF2‐associated meningioma cells (IOMM^NF2KD^, IOMM^518D^) at 4:1 for 2 days. The cytotoxicity of T cells was measured using flow cytometry based on the percentage of 7AAD^+^ cells in gated CFSE^+^ cells.

### 2.8 Western blotting

Total proteins were extracted from all treated cells using RIPA lysis buffer containing a protease and phosphatase inhibitor cocktail (PPLYGEN). The protein concentrations were measured with a BCA protein quantification assay (PPLYGEN). The proteins of each sample of 20–40 μg were loaded onto 12% SDS‐polyacrylamide gel electrophoresis (SDS–PAGE) and then transferred to PVDF membrane (PALL, USA). Then, the membranes were blocked with 5% nonfat milk at room temperature for 2 h, incubated with primary detection antibodies at 4°C overnight, and later incubated with HRP‐conjugated goat anti‐rabbit IgG/goat anti‐mouse IgG. The bands were detected using a Western‐Light Chemiluminescent Detection System (Millipore, USA). The relative density of immunoreactive bands was analyzed using ImageJ software (NIH, USA).

### 2.9 Quantitative RT–PCR

Total RNA was extracted with TRIzol (Transgene), and the concentrations and purity were determined by a Nanodrop 1000 spectrophotometer (Nanodrop Technologies). PrimeScript™ RT reagent Kit with gDNA Eraser (TAKARA, Kyoto, Japan) was used to prepare cDNA, and SYBR® Premix Ex Taq™ (TAKARA) was used to perform qRT‐PCR; GAPDH was used as a control for data normalization. Oligonucleotide primers for human PD‐L1 and GAPDH were obtained from Origene Co. (Rockville, MD). Gene expression was normalized to GAPDH expression using the 2^−ΔΔCT^ method.

### 2.10 In vivo experiments

Subcutaneous xenograft tumor models were established using NF2‐associated meningioma cells (IOMM^NF2KD^, IOMM^518D^) (1 × 10^6^ cells) in BALB/c nude mice. Twenty‐eight days later, the nude mice were randomly divided into 4 groups (*n* = 5/group). Mice were intraperitoneally administered 100 μL normal saline, 1.5 mg/kg durvalumab, 2 mg/kg rapamycin, or both. The tumor volume was assessed every 3 days. Tumor volume was calculated using the following formula: (the longest diameter) × (the shortest diameters)^2^/2. At the end of 21 days, the tumor xenografts were harvested and weighed.

### 2.11 Histology

Tissue samples and xenograft tumors were fixed in 4% paraformaldehyde, dehydrated in ethanol, embedded in paraffin, sectioned at 5 μm thickness and stained with hematoxylin and eosin.

### 2.12 Statistical analysis

Data are expressed as the median. For statistical data analysis, the Student's independent *t*‐test was used for normally distributed data. Mann–Whitney *U* test was used for non‐normally distributed data. We conducted a Shapiro–Wilk test to verify data distribution.

## 3 RESULTS

### 3.1 Infiltrating immune cell populations and expression of PD‐L1 in NF2‐associated meningioma

**FIGURE 1:**
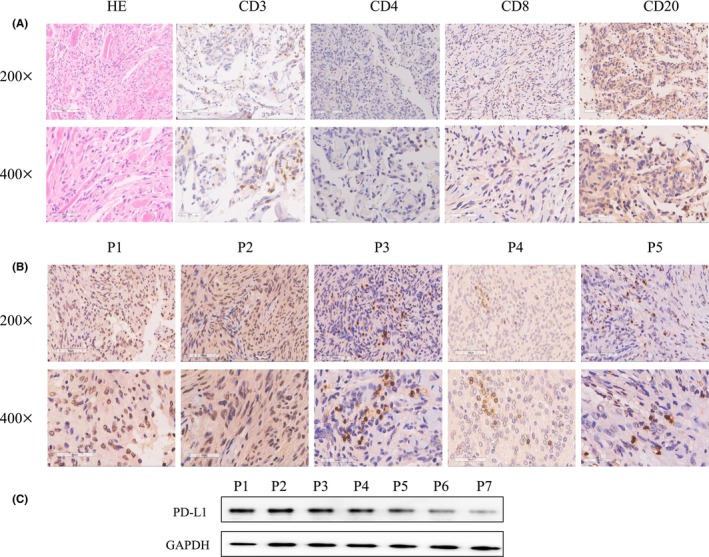
Tumor‐infiltrating lymphocytes and PD‐L1 expression in NF2‐associated meningioma. (A) IHC staining of CD3‐, CD8‐, CD4‐ and CD20‐positive lymphocytes in NF2‐associated meningiomas. (B) IHC staining of PD–L1 expression in NF2‐associated meningiomas at 200×/400× magnification. (C) Western blotting results of PD‐L1 expression in NF2‐associated meningiomas.

The type and location of lymphocytic infiltrate are associated with the tumor response to immune checkpoint inhibitors. We characterized NF2‐associated meningioma‐infiltrated lymphocytes using immunohistochemical staining with a specific antibody. Immunohistochemistry analysis confirmed the presence of CD3‐ and CD8‐positive T lymphocytes (13/13, 100%), CD4‐positive T lymphocytes (3/13, 23.08%), and CD20‐positive B lymphocytes (6/13, 46.15%) (Figure 1A).

PD‐L1 expression was also examined in NF2‐associated meningioma tissues. As reported before (10), we chose a rabbit monoclonal antibody (clone E1L3N) for IHC staining and Western blotting experiments because it is more specific and sensitive. Positive expression of PD‐L1 was observed in 38.46% (5/13) of NF2 meningiomas according to IHC results and 53.85% (7/13) of NF2 meningiomas according to Western blotting (Figure 1B,C).

Among PD‐L1‐positive patients, there were 6 males and 1 female (a high proportion of males). Among PD‐L1‐negative patients, there was 1 male and 5 females (a high proportion of females). The Fisher's test showed a *p*‐value of 0.029 for the sex distribution between the two groups, indicating a significant difference in gender. There were no significant differences in other clinical features, such as age (*p* = 0.951), tumor grade (*p* = 1.000), tumor volume (*p* = 0.962), and the number of meningiomas (*p* = 0.854), in these patients (Table S1). Male patients with meningiomas are more inclined to express PD‐L1, and immunomodulatory therapy may be more effective, but further validation is required with a larger sample size.

### 3.2 Downregulation of PD‐L1 inhibits NF2‐associated meningioma cell proliferation and promotes apoptosis

**FIGURE 2:**
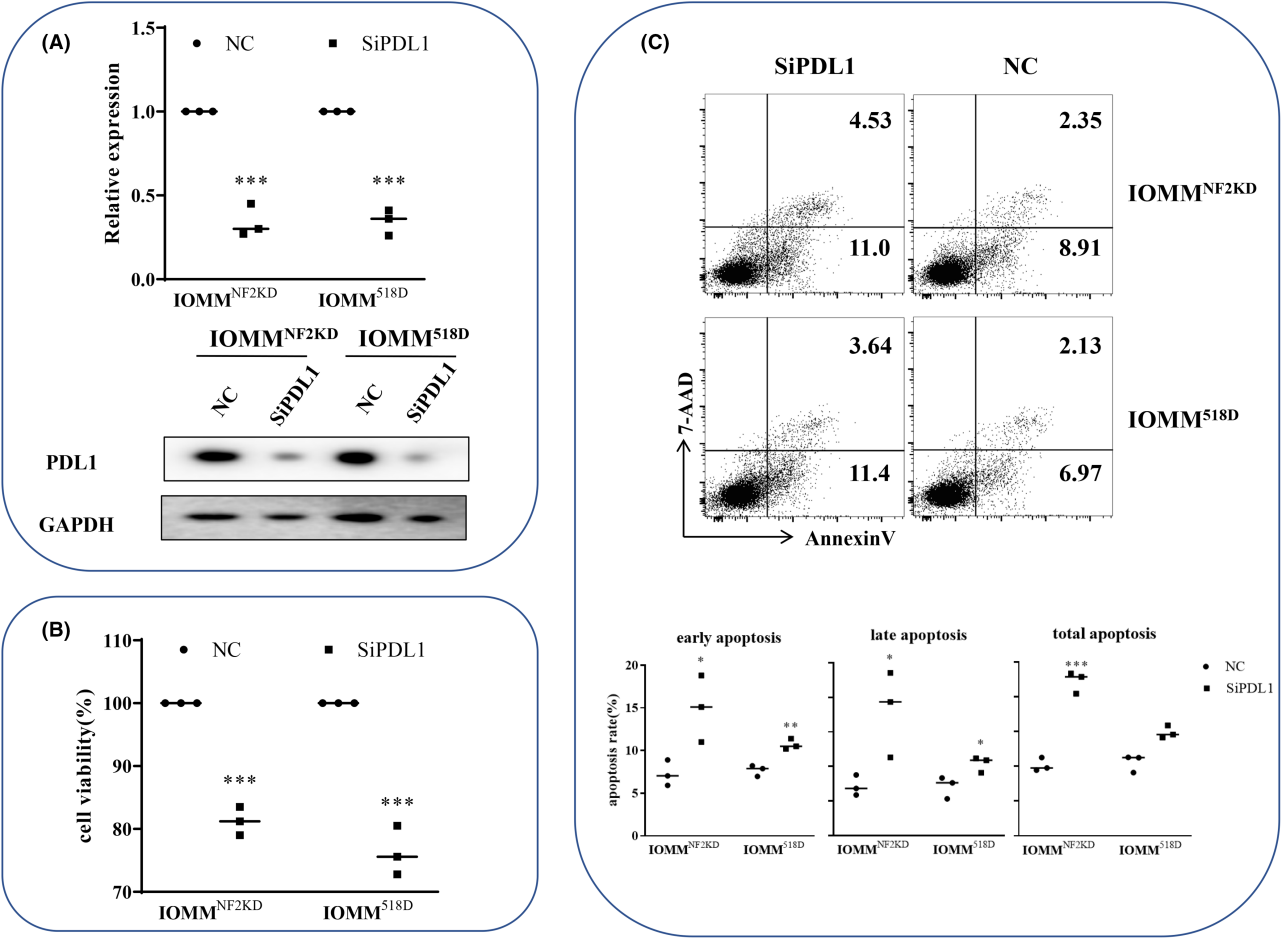
The effect of PD‐L1 downregulation on NF2‐associated meningioma cell proliferation and apoptosis. (A) Top: Relative expression of PD‐L1 after transfection with siPD‐L1 for 72 h (unpaired *t* test, vs. NC, *n* = 3). Bottom: Western blotting results showed PD‐L1 expression after transfection with siPD‐L1 for 72 h. (B) The cell viability of NF2‐associated meningioma cells after transfection with siPD‐L1 for 72 h was determined using the CCK‐8 assay. (C) Top: Flow cytometric analysis of NF2‐associated meningioma cells after transfection with siPD‐L1 for 72 h using Annexin V‐FITC/7‐AAD staining. Bottom: Statistical data of apoptosis rates. *p < 0.05, **p < 0.01, ***p < 0.005 compared to the NC group.

To better mimic NF2‐associated meningioma cells, we established a stable NF2 knockdown cell line by shRNA knockdown (IOMM^NF2KD^) and a stable 518D overexpression cell line by lentivirus infection (IOMM^518D^) in IOMM‐Lee cells. Furthermore, we designed a PD‐L1‐silencing construct and silenced PD‐L1 in NF2‐associated meningioma cells. After PD‐L1 knockdown in NF2‐associated meningioma cells, RT–PCR and Western blotting results indicated that the expression levels of PD‐L1 were significantly downregulated after transfection with si‐PD‐L1 compared with the NC group (Figure 2A). When PD‐L1 levels were downregulated by siPD‐L1 transfection in NF2‐associated meningioma cells, the CCK‐8 assay showed that the tumor cell growth rate was significantly reduced (Figure 2B), and both the early and late apoptosis rates were elevated (Figure 2C).

### 3.3 Downregulation of PD‐L1 suppresses the inhibitory effect of NF2‐associated meningioma cells on T‐cell activation and cytotoxicity

**FIGURE 3:**
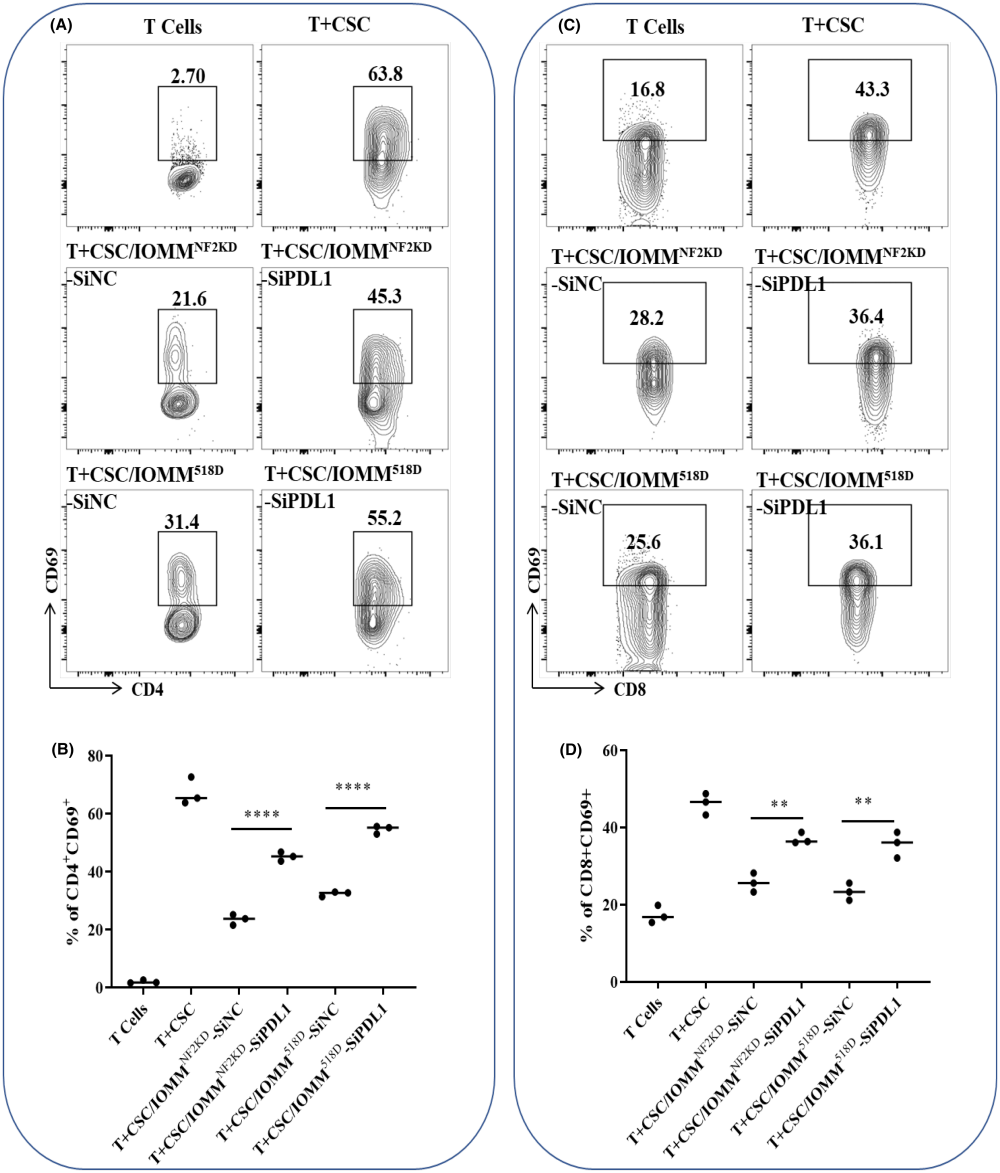
The effect of PD‐L1 downregulation on T‐cell activation. NF2‐associated meningioma cells transfected with siPD‐L1 were added at 1:4 to normal CSC‐stimulated CD3^+^ T cells. (A) Flow cytometric analysis of CD4^+^CD69^+^ cell percentages in the coculture system. (B) Statistical analysis of the flow cytometric results shown in (A), *n* = 3. (C) Flow cytometric analysis of CD8^+^CD69^+^ cell percentages in the coculture system. (D) Statistical analysis of the flow cytometric results shown in (C), *n* = 3. *p < 0.05, **p < 0.01, ***p < 0.005 compared to the NC group.

To determine the roles of PD‐L1 in T‐cell activation, T cells were separated from healthy donors (HDs) and stimulated with Cell Stimulation Cocktail and cocultured with either siPD‐L1‐ or siPD‐L1‐NC‐transfected NF2‐associated meningioma cells. The percentages of CD4^+^CD69^+^ and CD8^+^CD69^+^ cells were analyzed, and the results showed that when T cells were cocultured with siPD‐L1‐transfected NF2‐associated meningioma cells, the expression of CD69 on both CD4^+^ and CD8^+^ T cells was partly reduced compared with that in the siPD‐L1‐NC group (Figure 3).

**FIGURE 4:**
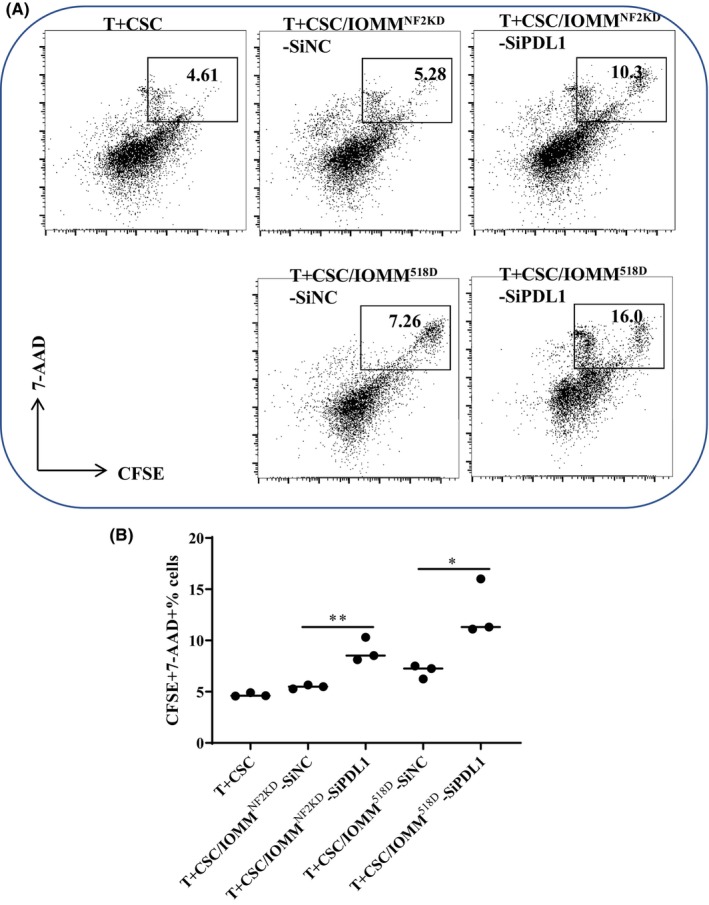
The effect of PD‐L1 downregulation on T‐cell cytotoxicity. NF2‐associated meningioma cells transfected with siPD‐L1 were added at 1:4 to normal CSC‐stimulated CD8^+^ T cells, and cytotoxicity was analyzed. (A) Flow cytometric analysis of CFSE^+^7AAD^+^ cell percentages in the coculture system. (B) Statistical analysis of the flow cytometric results shown in (A), *n* = 3. (*p < 0.05, **p < 0.01).

Furthermore, the effect of PD‐L1 downregulation in NF2‐associated meningioma cells on the influence of exT cell cytolytic activity was also evaluated. T cells were separated from HDs, stimulated with Cell Stimulation Cocktail and cocultured with either CFSE‐labeled siPD‐L1‐ or siPD‐L1‐NC‐transfected NF2‐associated meningioma cells. As expected, our results demonstrated that the ability of T cells to kill siPD‐L1‐transfected tumor cells was partly restored compared to that in the siPD‐L1‐NC group (Figure 4).

### 3.4 NF2 in PD‐L1 expression modulation

To assess the function of NF2 in modulating PD‐L1 expression, PD‐L1 expression was detected when NF2 was downregulated or phosphorylated. The results showed that the expression of PD‐L1 was increased at the same time as NF2 knockdown, suggesting that NF2 protein may regulate the expression of PD‐L1 through some mechanisms. Phosphorylation is an important way to regulate the function of NF2. We found that NF2 meningiomas with a higher degree of merlin phosphorylation also had higher PD‐L1 expression, which further verified the function of NF2 in regulating PD‐L1.

**FIGURE 5:**
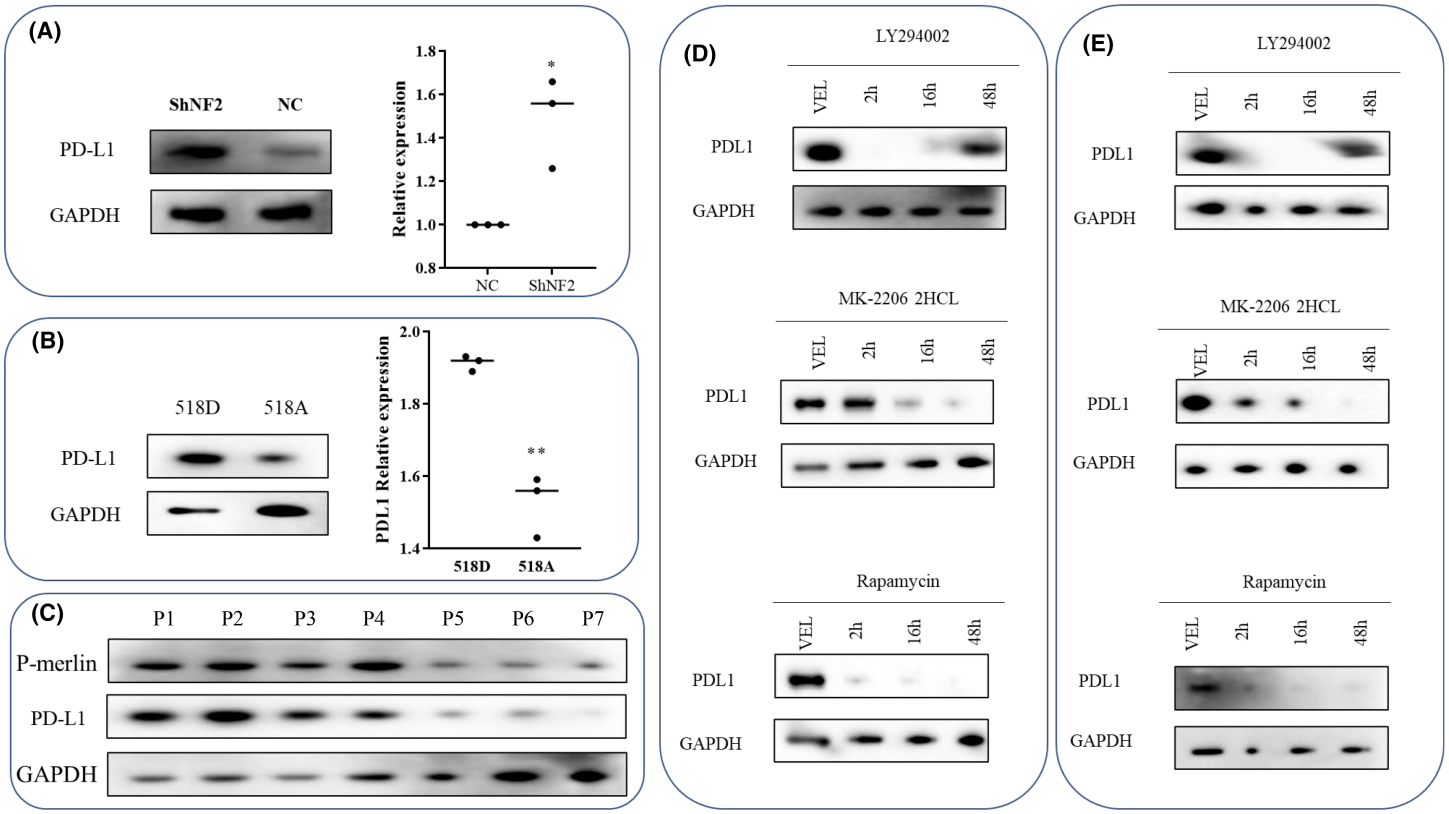
Regulation of PD‐L1 expression in NF2‐associated meningiomas. (A) Western blot analysis showed upregulation of PD‐L1 expression in IOMM‐Lee cells after knockdown of NF2 expression. *p < 0.05 compared to the NC/518D group. (B) Western blot analysis showed that phosphorylation of NF2 led to increased PD‐L1 expression in IOMM^518D^ cells compared with IOMM^518A^ cells. **p < 0.01 compared to the 518D group. (C) Western blotting results showed that NF2‐associated meningioma tissues with higher P‐merlin expression also had higher PD‐L1 expression. (D) IOMM^NF2KD^ cells were treated with 50 μmol/L of a PI3K inhibitor (LY294002), 2 μmol/L of an mTOR inhibitor (rapamycin), or 5 μmol/L of an AKT inhibitor (MK‐2206 2HCL), and the phosphorylation events downstream of AKT/mTOR were monitored by Western blot analysis. (E) IOMM^518D^ cells were treated with 50 μmol/L of a PI3K inhibitor (LY294002), 2 μmol/L of an mTOR inhibitor (rapamycin), or 5 μmol/L of an AKT inhibitor (MK‐2206 2HCL), and the phosphorylation events downstream of AKT/mTOR were monitored by Western blot analysis.

Merlin, a tumor suppressor protein encoded by the NF2 gene, plays an important role in maintaining cell membrane stability and regulating several cellular growth pathways. Loss of merlin leads to the abnormal activation of multiple pathways, and the PI3K–AKT–mTOR pathway is one of the most important pathways. To assess whether the PI3K–AKT–mTOR pathway regulates PD‐L1 expression in NF2 meningiomas, NF2‐associated meningioma cells were treated with inhibitors of PI3K (LY294002, 50 μmol/L), AKT (MK‐2206 2HCL, 5 μmol/L), and mTOR (rapamycin, 2 μmol/L). After 48 h of PI3K inhibitor (LY294002) treatment, recovery of PDL1 expression was observed, possibly due to its short half‐life (Figure 5). The results show that the inhibitors of PI3K, AKT, and mTOR decreased PD‐L1 expression in a time‐dependent manner, and mTOR inhibition could rapidly and persistently reduce PD‐L1 expression. In conclusion, these results indicate that inhibition of the PI3K–AKT–mTOR pathway decreases PD‐L1 expression.

### 3.5 The combination of rapamycin and an anti‐PD‐L1 antibody inhibits NF2‐associated meningioma cell growth in vivo

**FIGURE 6:**
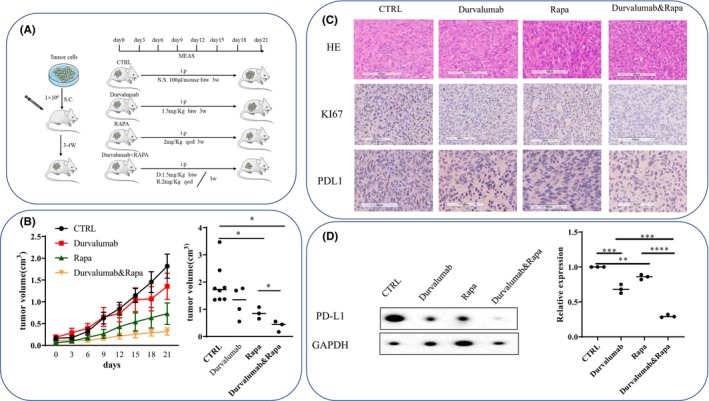
The combination of rapamycin and anti‐PD‐L1 antibody inhibits NF2‐associated meningioma cell growth in vivo. (A) Figure of the animal experimental design and schedule. (B) Representative graph of tumor volume measured every 3 days from the beginning of treatment. Volume of the tumors excised from treated and control mice after the mice were sacrificed. (C) Pathologic analysis of H&E, Ki67 and PD‐L1 staining of the tumors excised from the treated and control mice after 21 days of treatment. (D) Western blot analysis of PD‐L1 expression in the tumors excised from treated and control mice after 21 days of treatment. *p < 0.05, **p < 0.01, ***p < 0.005, ****p < 0.001 compared to the CTRL group.

Furthermore, we validated the roles of PD‐L1 downregulation in NF2‐associated meningioma progression in vivo. We constructed a BALB/C nude xenograft mouse model in which mice were transplanted with NF2‐associated meningioma cells. After 4 weeks, the combination of rapamycin and anti‐PD‐L1 therapy significantly reduced tumor volume compared with other groups. Rapamycin therapy alone also significantly decreased the tumor volume, whereas anti‐PD‐L1 antibody therapy alone had no effect. The combination therapy also decreased KI67 and PD‐L1 expression significantly compared with the other groups (Figure 6). These findings suggest that reduced PD‐L1 expression could contribute to antitumor efficacy.

## 4 DISCUSSION

In this study, we show that positive expression of PD‐L1 was observed in 38.46% (5/13), and CD3‐ and CD8‐positive T lymphocytes were present in 100% (13/13) of NF2‐associated meningiomas, indicating the possibility of anti‐PD‐L1 therapy for NF2‐associated meningiomas. Further results confirmed that downregulation of PD‐L1 could inhibit tumor cell proliferation, promote apoptosis, and suppress the inhibitory effect of tumor cells on T‐cell activation and cytotoxicity in NF2‐associated meningiomas. Further mechanistic studies revealed that downregulation or phosphorylation of NF2 may regulate PD‐L1 expression through the PI3K–AKT–mTOR pathway, with the mTOR inhibitor rapamycin having a rapid and persistent function in reducing PD‐L1 expression. The in vivo experimental results suggest that the anti‐PD‐L1 antibody may have a synergetic effect with the mTOR inhibitor in reducing tumor cell proliferation and that reduced PD‐L1 expression could contribute to antitumor efficacy.

The antitumor therapeutic effect of PD‐L1 is associated with the infiltration of lymphocytes and the concrete type.^15^, ^16^, ^17^ In this study, we first determined the infiltrating immune cell populations in NF2‐associated meningiomas. The results showed the presence of CD4‐positive T lymphocytes (3/13, 23.08%) and CD20‐positive B lymphocytes (6/13, 46.15%) in NF2‐associated meningiomas. The results also showed that all of the NF2‐associated meningiomas had CD3‐ and CD8‐positive T lymphocytes (13/13, 100%), which are considered indicators of a good PD‐L1 therapy response, providing a foundation for anti‐PD‐L1 therapy.

Next, we detected the expression of PD‐L1 in NF2‐associated meningiomas and found that positive expression of PD‐L1 was observed in 38.46% (5/13) of NF2‐associated meningiomas according to IHC and 53.85% (7/13) of NF2‐associated meningiomas according to Western blotting. Western blotting results showed a higher PD‐L1 expression rate due to its higher sensitivity, which may partly illustrate why some tumor patients with PD‐L1‐negative tumors could still have a significant response to a PD‐L1 inhibitor, which is probably related to sensitivity.

PD‐L1 is a key immune suppressor, and its expression is upregulated in numerous malignant tumor types and has been correlated with tumor progression and patient survival.^18^, ^19^, ^20^, ^21^ A previous study also reported that its expression in the tumor compartment contributed to tumor growth.^22^, ^23^ As we have confirmed the expression of PD‐L1 in NF2‐associated meningiomas, we next designed siRNAs and silenced PD‐L1. After PD‐L1 levels were downregulated by siPD‐L1 transfection in NF2‐associated meningioma cells, cell proliferation was significantly slowed, and the apoptosis rate was elevated, indicating that PD‐L1 may have important functions in promoting tumor formation and development. Similar results were reported in leukemia, non‐small cell lung cancer and melanoma.^22^, ^24^, ^25^, ^26^

PD‐L1 expression is an immune evasion mechanism that has been demonstrated in many tumors.^27^, ^28^, ^29^ PD‐L1 interacts with antigen‐presenting cells (APCs) or its receptor PD‐1 on T lymphocytes, leading to T lymphocyte activity inhibition, apoptosis or anergy.^30^, ^31^ Therefore, we next evaluated the effect of PD‐L1 downregulation on T‐cell activation and cytotoxicity. The results show that when T cells were cocultured with siPD‐L1‐transfected NF2‐associated meningioma cells, the expression of CD69 on both CD4^+^ and CD8^+^ T cells was partly restored, and the capacity of T cells to kill siPD‐L1‐transfected tumor cells was partly recovered, suggesting that targeting PD‐L1 could promote T‐cell activation, prevent tumor‐immune escape and help T cells kill tumor cells in NF2‐associated meningioma.

The above results indicated that PD‐L1 expression on the cell membrane surface of NF2‐associated meningiomas is an important cause of tumor development and lymphocyte dysfunction; identifying the potential pathways that regulate PD‐L1 expression may be helpful for restoring the function of tumor‐infiltrating lymphocytes and inhibiting tumor progression. The clinical benefit also supports efforts to study the mechanism that regulates tumor PD‐L1 expression and therapeutic interventions to decrease PD‐L1 levels.^32^ Multiple mechanisms, such as MAPK, NF‐κB, and AKT–mTOR pathway regulation, can contribute to intrinsic PD‐L1 expression in tumors.^33^, ^34^, ^35^, ^36^, ^37^, ^38^, ^39^, ^40^ As the PI3K–AKT–mTOR pathway plays a key role in NF2,^41^, ^42^, ^43^ we hypothesized that this pathway may be responsible for controlling PD‐L1 expression in NF2‐associated meningiomas. The results confirmed our hypothesis that inhibition of PI3K, Akt, or mTOR using different pharmaceutical inhibitors decreased PD‐L1 expression in a time‐dependent manner, with rapamycin (mTOR inhibitor) rapidly and persistently reducing PD‐L1 expression, indicating that the pathway plays an important role in tumor progression, not only by influencing tumor cell characteristics but also by altering the immune system in the tumor microenvironment. These results also suggest that combining PI3K–AKT–mTOR inhibitors with PD‐L1‐targeting therapy and optimizing these combinations may improve the treatment effect. We also identified the effect of rapamycin on the expression of β‐TrCP in NF2‐associated meningioma cells. The results show that the expression of β‐TrCP decreases in NF2‐associated meningioma cells after treatment with rapamycin (2 μmol/L 48 h), suggesting that the stability of PDL1 can also be regulated by inhibiting the mTOR pathway. The results are shown in Figure S1. Additionally, we measured the expression level of EGFR in NF2‐assoicated meningioma cells. The experimental results showed that, compared to the control group, NF2‐associated meningioma cells exhibited increased expression of EGFR, indicating enrichment of EGFR in NF2‐associated meningioma cells. Results also revealed that EGF (100 ng/mL, 48 h), an agonist of EGFR signaling, could remarkably upregulate PD‐L1 protein expression in NF2‐associated meningioma cells, demonstrating that EGF can induce an increase in PDL1 expression. The results are shown in Figure S2.

In vivo experimental results showed that anti‐PD‐L1 antibody combined with mTOR inhibitor could effectively reduce tumor proliferation with a significant reduction in PD‐L1 and KI67 expression compared with that in any other group, suggesting that anti‐PD‐L1 may have a synergetic effect with mTOR inhibitors, and the significant effect in reducing tumor volume may be due to PD‐L1 downregulation. Although anti‐PD‐L1 antibody therapy alone had no effect, it may regulate the anti‐PD‐L1 dosage or the absence of T cells in nude mice. We will further disclose this mechanism in follow‐up experiments.

In summary, we demonstrated that PD‐L1 is heterogeneously expressed in NF2‐associated meningiomas, the PI3K–AKT–mTOR pathway regulates its expression, and targeting PD‐L1 could be helpful for recovering the function of tumor‐infiltrating lymphocytes and inducing apoptosis to inhibit tumor proliferation. Dissecting the mechanisms of the PD‐L1‐driven tumorigenesis of NF2‐associated meningioma will help to improve our understanding of the mechanisms underlying tumor progression and could facilitate the further refinement of current therapies to improve the treatment of NF2 patients.

## CONFLICT OF INTEREST STATEMENT

The authors declare that they have no competing interests.

## INFORMED CONSENT

Informed consent was obtained from all individual participants included in the study.

## Supporting information


Appendix S1

## Data Availability

The authors confirm that the data supporting the findings of this study are available within the article [and/or] its supplementary materials.
