# Supplementary material for: PD‐L1 regulates tumor proliferation and T‐cell function in NF2‐associated meningiomas
Source: CNS Neurosci Ther. 2024 Jun 3;30(6):e14784. doi: 10.1111/cns.14784 (PMC11145367; doi:10.1111/cns.14784)
Supplement: Supplementary file 1 — Appendix S1 [file CNS-30-e14784-s001.docx]

**sFig1**


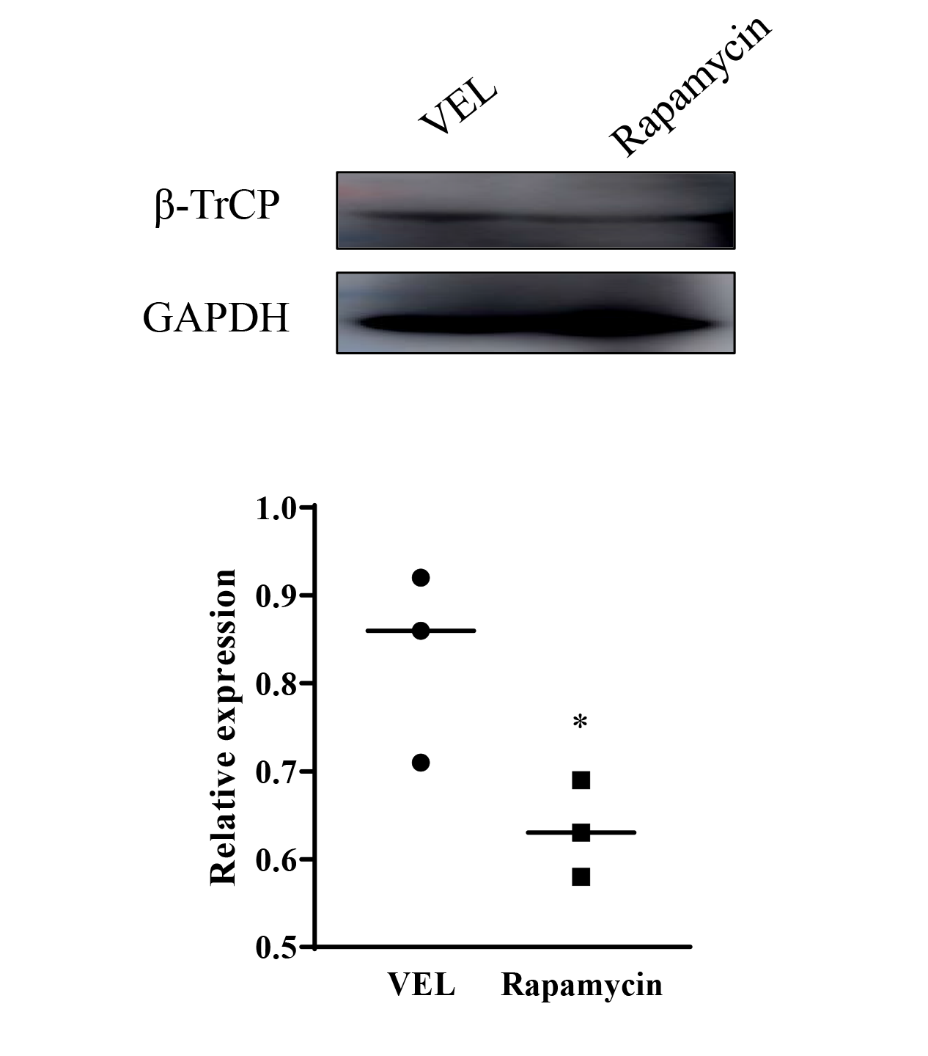


**sFig1 Rapamycin decrease expression of β-TrCP in NF2-associated meningioma cells** **p*<0.05 compared to the VEL group.

**sFig2**


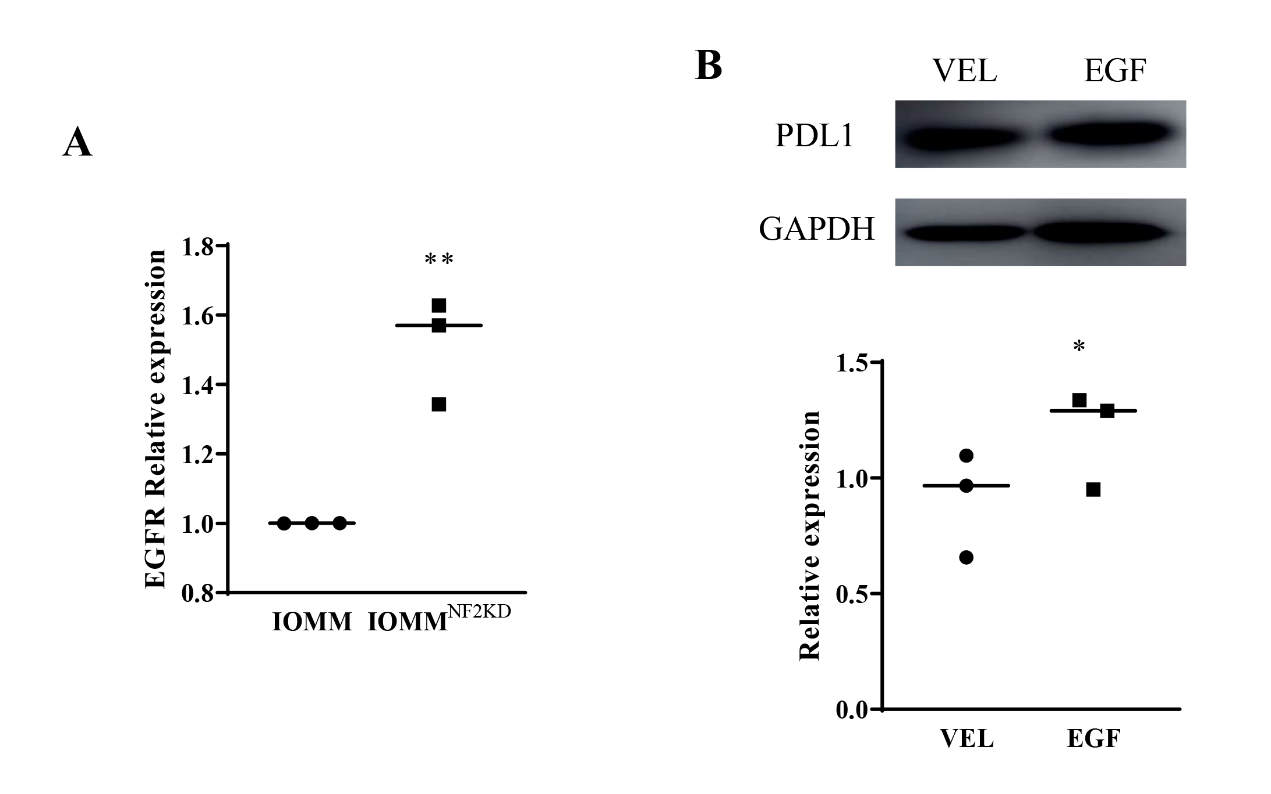


**sFig2: EGF could upregulate PD-L1 protein expression in NF2-associated meningioma cells**

A: NF2-associated meningioma cells exhibited increased expression of EGFR. ***p*<0.01 compared to the IOMM group.

B: EGF (100 ng/mL, 48 h), an agonist of EGFR signaling, could upregulate PD-L1 protein expression in NF2-associated meningioma cells. **p*<0.05 compared to the VEL group.

| Characteristics | PD-L1  positive | PD-L1  negative | Test value | P value |
| --- | --- | --- | --- | --- |
| Gender |  |  | Fisher’s precise test | 0.029* |
| Male | 6 | 1 |  |  |
| Female | 1 | 5 |  |  |
| Patient Age (years) | 34.6±18.2 | 35.2±15.1 | t=0.063 | 0.951 |
| Tumor volume (CM^3^) | 40.6±36.4 | 41.8±49.6 | t=0.048 | 0.962 |
| Meningioma number | 3.1±1.6 | 3.3±2.1 | t=0.189 | 0.854 |
| Meningioma grade |  |  | Fisher’s precise test | 1.000 |
| 1 | 4 | 4 |  |  |
| 2 | 3 | 2 |  |  |
| Tumor Location |  |  | Fisher’s precise test | 1.000 |
| Supratentorial | 5 | 4 |  |  |
| Infratentorial | 2 | 2 |  |  |

**Supplemental table 1**

**Statistical Results of Meningioma’s Characteristics**
